# Population-based centile curves for triceps, subscapular, and abdominal skinfold thicknesses in Polish children and adolescents—the OLAF study

**DOI:** 10.1007/s00431-012-1717-5

**Published:** 2012-03-21

**Authors:** Maciej Jaworski, Zbigniew Kułaga, Paweł Płudowski, Aneta Grajda, Beata Gurzkowska, Ewelina Napieralska, Anna Świąder, Huiqi Pan, Mieczysław Litwin, the Olaf Study Group

**Affiliations:** 1 Department of Biochemistry and Experimental Medicine, The Children’s Memorial Health Institute, Warsaw, Poland; 2 Public Health Division, The Children’s Memorial Health Institute, Warsaw, Poland; 3 Anthropology Division, Department of Pediatrics, The Children’s Memorial Health Institute, Warsaw, Poland; 4 MRC Centre of Epidemiology for Child Health, UCL Institute of Child Health, London, UK; 5 Department of Nephrology and Arterial Hypertension, The Children’s Memorial Health Institute, Warsaw, Poland; 6 Department of Research, The Children’s Memorial Health Institute, Warsaw, Poland

**Keywords:** Triceps, Subscapular, Abdominal skinfold thickness, Population-based values, Children, Polish

## Abstract

Skinfold thicknesses are used as valid anthropometric indicators of regional body fatness. Actual population-based values for skinfold thicknesses for Polish children are not available. The purpose of this study was to provide population-based values for triceps, subscapular, and abdominal skinfold thicknesses in healthy children and adolescents. A total number of 17,416 boys and girls aged 6.5–18.5 years, randomly selected from whole Polish population of children and adolescents, were enrolled in the study. Skinfold thicknesses (triceps, subscapular, and abdominal) were measured using Harpenden skinfold caliper. All measurements were taken after the training of participating investigators. The *LMS* method was used to fit percentile curves across age for each skinfold. *Q* tests for fit were used to assess the global goodness of fit of our final models. The study shows for the first time smoothed population-based values of body fat distribution indices for Polish children and adolescents 7–18 years of age. Reported skinfold centiles are higher compared to previously established for Warsaw children and very close to the actual US data. *Conclusion* Our study provided for the first time population-based values for skinfold thicknesses evaluation in a way allowing to calculate reliable *Z* scores. The early detection of abnormal fat stores, using our population-based values and respective *Z* scores, may be now implemented for practice.

## Introduction

Skinfold thicknesses evaluation, due to its low cost and noninvasive procedure, is one of the most widely used anthropometric methods for assessment of nutritional status during growth and maturation period [7, 10, 23, 25]. Excess fat, as assessed using skinfold thicknesses, is associated with abnormal concentrations of triglycerides, increased low-density lipoprotein cholesterol, reduced high-density lipoprotein cholesterol, and insulin resistance; all factors markedly increasing risk for hypertension, metabolic syndrome, and cardiovascular disease [8, 21].

Skinfold measurements have low precision error but should be done by well-trained personnel [23]. In practice, the magnitude of precision error largely depends on the skills of examiner conducting examinations, however is low compared to variability observed between subject [24, 25]. Skinfold thickness assessments are also utilized to identify individual body somatotype, enabling estimations of body density and lean body mass [14, 22, 25].

Reliable determination of references (medians and percentiles) for a given skinfold thicknesses requires a large set of healthy subjects [1]. Previous methodologies used empirical percentiles according to sex and age groups. Recently, the reference values based on smoothed curves across the age groups are favored. The most preferred method to establish references is *LMS* method. Box–Cox transformation allows transformation of data to normality by a suitable power transformation. Standardized *Z* scores can be calculated from transformed data [4, 5].

Actual population-based references for skinfold thicknesses are not available for Polish children, and local data are limited to urban regions, including the cities of Warsaw and Cracow [2, 18]. Further, the data from Warsaw study were collected more than a decade ago, comprised only children living in the city area, and reference data were developed using outdated statistical procedures that not allowed to calculate *Z* scores. The references from Cracow study were collected 9 years ago and as well as in Warsaw comprised only urban children. Although, the Cracow data were established by *LMS* method, Box–Cox transformation power (*L* value), median (*M*), and generalized coefficient of variation (*S* value) were not published; therefore, calculation of *Z* scores was not available. Since skinfold thickness evaluation is still considered as useful method for assessment of nutritional status during growth and because actual and appropriately established references are missing for Polish population, the purpose of this study was to provide population-based values for triceps, subscapular, and abdominal skinfold thicknesses for children and adolescents aged 7–18 years.

## Material and methods

### Population

The analyzed data were collected in the course of the OLAF study in which the reference blood pressure ranges were elaborated for Polish children and adolescents. The study sample comprised data collected between November 2007 and November 2009. Study participants were randomly selected using two-stage sampling. Primary units (schools) were sampled from an all-schools-in-Poland sampling frame; sampling was stratified by urban/rural area. In the second stage, all pupils in the required age range within the sampled schools comprised the sampling frame. Pupils in schools were selected for the survey by stratified random sampling, the stratification variables being classes. The medical history of the study participants, including past and present diseases, as well as medications used, was taken from the parents. The general health status of each subject was assessed by a physician. Exclusion criteria was as follows: infection with diarrhea leading dehydration, absence of right upper limb or phocomelia, dwarfism, cachexia, Cushing syndrome, renal failure, heart failure, hepatic failure, etc., organ transplantation, systemic diseases, malignant cancer, Turner syndrome, Down syndrome, etc., systemic glucocorticoids use, and pregnancy.

All subjects and their parents (in the case of subjects under 18 years of age) gave their informed consent to participate in the study (subjects over 16 years of age and parents gave written consent). Ethical approval was obtained from Ethical Committee of The Children’s Memorial Health Institute before the study commenced.

A total number of 17,416 boys and girls aged 6.5–18.5 years with measured at least one skinfold thickness were enrolled in the study. The response rate was 0.71. We excluded small number of outliers (7 for triceps, 21 for abdominal, and 13 for subscapular) following ±5 standard deviation criteria as was done by other authors [1, 11]. Characteristics of studied group were presented in Tables 1 and 2.

**Table 1:** Number of subjects by sex and age

Age group years	Sex	
	Female	Male
6.5–7.5	392	454
7.5–8.5	701	729
8.5–9.5	703	742
9.5–10.5	753	658
10.5–11.5	683	672
11.5–12.5	642	641
12.5–13.5	657	646
13.5–14.5	775	678
14.5–15.5	730	735
15.5–16.5	829	700
16.5–17.5	1,075	813
17.5–18.5	1,155	853
Sub-total	9,095	8,321
Total	17,416

**Table 2:** Characteristic of studied group

	Female			Male		
	Median	Min.	Max.	Median	Min.	Max.
Height (cm)	157.9	106.7	185.0	158.8	109.8	200.1
Weight (kg)	47.6	14.2	119.6	48.4	16.0	128.0
Body mass index (kg/m^2^)	18.9	11.6	46.3	18.8	11.9	45.1

### Measurements

The OLAF study (PL0080) was carried out in 416 schools (325 cities, countries, and villages) covering all regions of Poland. Measurements were conducted in school nurses’ offices from 8:00 a.m. to 3:00 p.m.; room temperature was in range 20–26°C. All measurements were taken by centrally trained staff: anthropologists, nurses, public health professionals, and physicians using the standard and calibrated equipment from the same manufacturer.

Body height was measured in the standing position using stadiometer (SECA 214). Body weight was measured using medical scale (Radwag WPT 100/200). Body mass index was calculated as body weight divided by height in meters squared. The exact age of each participant was calculated from birth and observation dates.

Skinfold thickness measurements were performed by lifting a fold of skin and subcutaneous fat away from the underlying muscle and bone. Each skinfold thickness was measured in duplicate with Harpenden skinfold caliper. When a difference between the first and the second measurement exceeded 6 mm, a third measurement was taken. The triceps skinfold was lifted parallel to the long axis of the body, midway on the back of the hanging freely right upper arm. The subscapular skinfold was lifted horizontally below the tip of right scapula. The abdominal skinfold was lifted diagonal midway between umbilicus and right anterior superior iliac spine.

All measurements were taken by centrally trained staff. The training consisted of workshops for study teams, during which the standardized measuring technique was presented (lecture, visuals) and taught (practical exercises). Following the workshop, in-the-field standardization sessions were conducted according to the standardization protocol. Reliability of skinfold thickness measurements between the trainer and the study staff was recorded. After 10 months of conducting the study, re-training of study teams was carried out.

### Statistics

The *LMS* method [4] was used to fit percentile curves across age for each skinfold. LMSchartmaker v. 2.43 (Medical Research Council, UK) [20] was used to derive the smoothed percentiles. The *LMS* method uses polynomial splines to fit smoothed curves: *L* (Box–Cox transformation power), *M* (median), and *S* (generalized coefficient of variation) across age by maximized penalized likelihood [5]. The smoothed percentile estimates and the *L*, *M*, and *S* parameters were derived from raw data, separately for each skinfold and sex, in a single-stage modeling. *Q* tests for fit [19] were used to assess the global goodness of fit of our final models.

## Results

**Fig. 1:**
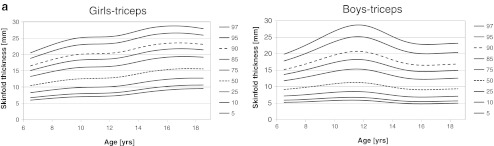

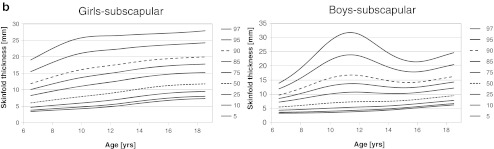

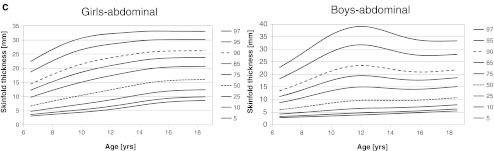
Smoothed percentile curves for skinfold thicknesses for boys and girls across age. *Dotted line* (median) and *dashed line* (90th percentile) were used for better readability

The smoothed percentiles for triceps, subscapular, and abdominal skinfold thicknesses are presented in Fig. 1 for boys and girls, separately. In girls, median for triceps skinfold thickness increased from 10.4 mm (6.5 years) to 15.6 mm (18.5 years) with plateau between 10 and 12 years and maximum (15.6 mm) at age 17.5 years. Ninety-seventh percentile increased from 20.5 mm at age 6.5 years to 27.9 at age 18.5 years with maximum (28.7 mm) at age 16.5 years. Plateau occurred between 10.5 and 12.0 years. Median for subscapular skinfold thickness increased from 10.0 to 11.8 mm for 6.5 and 18.5 years, respectively, and 97th percentile increased from 19.0 to 27.9 mm for the same ages. Median abdominal skinfold thickness increased from 6.6 mm (6.5 years) to 16.0 mm (18.5 years), and 97th percentile increased from 22.4 to 33.0 mm for the corresponding ages, respectively.

In boys, age-dependent changes and shape of percentile curves were more complex. Local maximums at age 11–12 years occurred for most of the percentiles of all skinfold thicknesses. For triceps skinfold thickness median increased from 9.2 mm (6.5 years) to 11.3 mm (11.5 years), then decreased to 9.1 mm (16.0 years), and then slightly increased to 9.4 mm at age 18.5 years. Ninety-seventh percentile increased from 19.9 mm (6.5 years) to 28.8 mm (11.5 years), then decreased to 22.8 mm (16.5 years), and then slightly increased to 23.2 mm at age 18.5 years. For subscapular skinfold thickness, median increased from 5.5 mm (6.5 years) to 9.5 mm (18.5 years), whereas 97th percentile increased from 13.9 mm (6.5 years) to 31.8 mm (11.5 years), then decreased to 21.4 mm (16.0 years), and then increased to 24.7 mm (18.5 years). For abdominal skinfold thickness, median increased from 5.9 mm (6.5 years) to 9.7 mm (12.5 years), then very slightly decreased to 9.5 mm (14.5 years), and then increased to 10.7 mm at age 18.5 years. Ninety-seventh percentile increased from 22.8 (6.5 years) to 39.1 mm (12.0 years) and then decreased to 33.3 mm at age 18.5 years. Smoothed *LMS* parameters across age for all measured skinfold thicknesses by sex are shown in Tables 3, 4, and 5.

**Table 3:** *LMS* parameters for triceps skinfold thickness by sex and age and equivalent degrees of freedom

Age	Girls			Boys		
	*L* (edf 1)	*M* (edf 6)	*S* (edf 3)	*L* (edf 1)	*M* (edf 6)	*S* (edf 3)
6.5	−0.07824	10.451	0.3492	−0.3034	9.146	0.3676
7.0	−0.07824	10.836	0.3510	−0.3034	9.346	0.3758
7.5	−0.07824	11.209	0.3529	−0.3034	9.562	0.3840
8.0	−0.07824	11.566	0.3547	−0.3034	9.806	0.3922
8.5	−0.07824	11.893	0.3564	−0.3034	10.076	0.4000
9.0	−0.07824	12.177	0.3578	−0.3034	10.361	0.4075
9.5	−0.07824	12.401	0.3589	−0.3034	10.647	0.4143
10.0	−0.07824	12.543	0.3596	−0.3034	10.906	0.4203
10.5	−0.07824	12.617	0.3596	−0.3034	11.122	0.4252
11.0	−0.07824	12.660	0.3590	−0.3034	11.269	0.4292
11.5	−0.07824	12.707	0.3578	−0.3034	11.308	0.4321
12.0	−0.07824	12.799	0.3558	−0.3034	11.208	0.4341
12.5	−0.07824	12.985	0.3532	−0.3034	10.976	0.4353
13.0	−0.07824	13.280	0.3500	−0.3034	10.639	0.4357
13.5	−0.07824	13.665	0.3462	−0.3034	10.244	0.4354
14.0	−0.07824	14.090	0.3421	−0.3034	9.847	0.4347
14.5	−0.07824	14.498	0.3377	−0.3034	9.508	0.4336
15.0	−0.07824	14.848	0.3332	−0.3034	9.258	0.4322
15.5	−0.07824	15.130	0.3286	−0.3034	9.110	0.4306
16.0	−0.07824	15.353	0.3241	−0.3034	9.056	0.4290
16.5	−0.07824	15.518	0.3197	−0.3034	9.074	0.4275
17.0	−0.07824	15.618	0.3155	−0.3034	9.136	0.4260
17.5	−0.07824	15.649	0.3113	−0.3034	9.216	0.4245
18.0	−0.07824	15.623	0.3072	−0.3034	9.292	0.4232
18.5	−0.07824	15.569	0.3031	−0.3034	9.358	0.4218

*L* Box–Cox transformation power, *M* median, *S* generalized CV, *edf* equivalent degrees of freedom

**Table 4:** *LMS* parameters for subscapular skinfold thickness by sex and age and equivalent degrees of freedom

Age	Girls			Boys		
	*L* (edf 3)	*M* (edf 5)	*S* (edf 4)	*L* (edf 3)	*M* (edf 6)	*S* (edf 5)
6.5	−0.6912	5.955	0.4249	−0.6445	5.493	0.3712
7.0	−0.6748	6.249	0.4313	−0.6382	5.663	0.3932
7.5	−0.6581	6.538	0.4377	−0.6322	5.845	0.4153
8.0	−0.6412	6.821	0.4437	−0.6270	6.045	0.4373
8.5	−0.6241	7.099	0.4491	−0.6232	6.263	0.4581
9.0	−0.6068	7.370	0.4534	−0.6216	6.490	0.4767
9.5	−0.5901	7.632	0.4560	−0.6224	6.710	0.4917
10.0	−0.5745	7.883	0.4567	−0.6263	6.903	0.5019
10.5	−0.5606	8.128	0.4551	−0.6340	7.066	0.5068
11.0	−0.5490	8.374	0.4513	−0.6460	7.204	0.5060
11.5	−0.5401	8.630	0.4456	−0.6624	7.312	0.4994
12.0	−0.5344	8.907	0.4379	−0.6829	7.380	0.4874
12.5	−0.5323	9.211	0.4289	−0.7069	7.414	0.4710
13.0	−0.5339	9.540	0.4190	−0.7337	7.434	0.4515
13.5	−0.5391	9.881	0.4087	−0.7627	7.464	0.4302
14.0	−0.5475	10.216	0.3983	−0.7929	7.520	0.4083
14.5	−0.5588	10.532	0.3881	−0.8233	7.620	0.3874
15.0	−0.5723	10.813	0.3786	−0.8532	7.764	0.3687
15.5	−0.5873	11.052	0.3702	−0.8821	7.951	0.3530
16.0	−0.6034	11.249	0.3632	−0.9098	8.180	0.3412
16.5	−0.6203	11.404	0.3578	−0.9364	8.443	0.3335
17.0	−0.6377	11.521	0.3538	−0.9619	8.717	0.3292
17.5	−0.6556	11.611	0.3506	−0.9866	8.985	0.3268
18.0	−0.6739	11.688	0.3480	−1.0109	9.248	0.3246
18.5	−0.6925	11.763	0.3454	−1.0350	9.511	0.3222

*L* Box–Cox transformation power, *M* median, *S* generalized CV, *edf* equivalent degrees of freedom

**Table 5:** *LMS* parameters for abdominal skinfold thickness by sex and age and equivalent degrees of freedom

Age	Girls			Boys		
	*L* (edf 3)	*M* (edf 5)	*S* (edf 4)	*L* (edf 4)	*M* (edf 5)	*S* (edf 4)
6.5	−0.3106	6.629	0.5389	−0.4553	5.902	0.5367
7.0	−0.2791	7.191	0.5422	−0.4239	6.241	0.5525
7.5	−0.2475	7.750	0.5454	−0.3930	6.591	0.5685
8.0	−0.2159	8.301	0.5481	−0.3629	6.964	0.5842
8.5	−0.1845	8.841	0.5502	−0.3345	7.359	0.5991
9.0	−0.1537	9.371	0.5510	−0.3092	7.773	0.6122
9.5	−0.1243	9.886	0.5499	−0.2876	8.190	0.6227
10.0	−0.0970	10.382	0.5463	−0.2703	8.588	0.6298
10.5	−0.0722	10.868	0.5401	−0.2588	8.947	0.6331
11.0	−0.0503	11.352	0.5312	−0.2548	9.250	0.6326
11.5	−0.0313	11.838	0.5199	−0.2592	9.478	0.6282
12.0	−0.0155	12.328	0.5066	−0.2713	9.615	0.6201
12.5	−0.0029	12.826	0.4919	−0.2897	9.668	0.6088
13.0	0.0063	13.328	0.4766	−0.3125	9.658	0.5952
13.5	0.0123	13.825	0.4614	−0.3379	9.613	0.5799
14.0	0.0153	14.301	0.4466	−0.3639	9.563	0.5639
14.5	0.0157	14.731	0.4327	−0.3884	9.537	0.5480
15.0	0.0138	15.089	0.4203	−0.4100	9.549	0.5330
15.5	0.0102	15.369	0.4097	−0.4271	9.607	0.5197
16.0	0.0050	15.579	0.4011	−0.4385	9.717	0.5086
16.5	−0.0016	15.732	0.3947	−0.4439	9.875	0.4996
17.0	−0.0092	15.834	0.3900	−0.4444	10.066	0.4926
17.5	−0.0174	15.896	0.3866	−0.4412	10.271	0.4871
18.0	−0.0260	15.939	0.3840	−0.4358	10.483	0.4825
18.5	−0.0349	15.979	0.3816	−0.4295	10.698	0.4781

*L* Box–Cox transformation power, *M* median, *S* generalized CV, *edf* equivalent degrees of freedom

## Discussion

This study is the first and the largest study of skinfold thicknesses based on a randomly selected sample of 17,416 boys and girls aged 6.5–18.5 years from both urban and rural areas of Poland. The study enabled to calculate and provide a reference data for skinfold thicknesses, as noninvasive estimators of fat stores in Polish children and adolescents aged 7–18 years.

As expected, our results revealed gender-related differences between boys and girls for triceps, subscapular, and abdominal skinfold thicknesses. In girls, percentile curves showed constant increase with age. In boys, subscapular, triceps, and abdominal skinfold thicknesses also increased with age; however, in contrast to girls, maximum values were noted in groups aged 11–12 year and then skinfold thicknesses returned to the values preceding the pubertal spurt. The same finding was already reported in previous studies from Warsaw [18], Cracow [2], and in other populations; however, the phenomenon was mostly evident for triceps skinfold thickness and in less extent for subscapular and abdominal skinfolds [1, 6, 9, 15–17]. Further, our study showed higher percentile and median values than previously reported in Warsaw [18] and Cracow studies [2]. The highest differences were observed for 97th percentile and ranged from 4 to 18 mm for peaks of skinfold thicknesses and from 2 to 13 mm for minimums. The increased skinfold thicknesses observed in our study highly likely reflected a secular trend in obesity noted during the last decades in Polish population [3, 12].

Furthermore, skinfold thicknesses noted in our study appeared higher than in Dutch [9] and Turkish [17] population. However, the Dutch study was conducted in years 1979–1980, when secular trends in obesity were considered as not very evident [9]. Although the Turkish data were collected in 2005 year, the study comprised population with very low prevalence of obesity [17]. To our knowledge, the highest normal skinfold thicknesses so far reported in literature were established in a representative sample of Spanish adolescents aged 13–18 years [16]. Nationally representative percentiles for US children and adolescents presented only slightly lower values [1, 6, 15] than Spanish data. Our study results appeared similar to US data.

The main limitation of our study is related to its cross-sectional design. Body fat indices in growing children should be rather obtained in longitudinal studies showing longitudinal changes in individual growth and development.

The strength of our study was that examined data were sampled in a random manner from whole population of Polish children and adolescents. Further, intra-observer as well as inter-observer measurement errors were considered as low and satisfactory (data not shown due to large number of investigators participating in this survey), and collected data were verified and checked for outliers. Moreover, the total number of subjects (17,416 boys and girls) allowed reliable determination of medians and percentiles [1, 4]. Finally, our data based on a nationally representative sample of Polish children and adolescents enabled for the first time precise evaluation of skinfold thicknesses using *Z* scores. In agreement with others [4, 13], we strongly recommend the calculation of *Z* scores corresponding to the percentile rankings, using the following formula: *Z* = {(skt/*M*)*L* − 1}/*LS*, where skt indicates actual skinfold thickness. In the case of *L* = 0, as noted for abdominal skinfold thickness in girls (Tables 3, 4, and 5), *Z* score value should be calculated as: *Z* = log(skt/*M*)/*S*. The last formula should be also used for *L* between −0.01 and +0.01 [4].

In conclusion, our study based on randomly selected sample of 17,416 healthy children and adolescents from Poland provide for the first time population-based values for skinfold thicknesses evaluation in a way allowing to calculate reliable *Z* scores. In consequence, the early detection of abnormal fat stores, using our data and respective *Z* scores, may be now implemented for everyday clinical practice.
